# Extensive Longitudinal Transverse Myelitis after Influenza A Virus Infection in a Patient with Systemic Lupus Erythematosus

**DOI:** 10.1155/2022/9506733

**Published:** 2022-01-07

**Authors:** Suheiry Márquez, Luis M. Vilá

**Affiliations:** Division of Rheumatology, University of Puerto Rico Medical Sciences Campus, San Juan, Puerto Rico

## Abstract

Transverse myelitis (TM) is a rare complication seen in 1–2% of patients with systemic lupus erythematosus (SLE). Viral infections may cause TM in these patients by causing a dysregulation of their immune system. We report a 30-year-old woman with SLE who had influenza A and a few days later developed urinary retention, bilateral lower extremity paralysis, upper extremity weakness, and optic nerve and macular edema. Magnetic resonance imaging showed C4-T12 hyperintense lesions consistent with TM. She was treated with intravenous methylprednisolone 1 g daily for 3 days and then 6 cycles of monthly intravenous cyclophosphamide. This treatment was followed by oral prednisone. She had a remarkable clinical response. Visual acuity improved to her baseline, and muscle strength almost fully recovered. Clinicians should be aware that viral infections, including influenza, may induce TM. This case highlights the importance of early recognition and prompt treatment with immunosuppressive drugs in such cases.

## 1. Introduction

Transverse myelitis (TM) is an infrequent, severe, and devastating immune-mediated neurologic disorder characterized by the sudden onset of motor, sensory, and autonomic nervous system dysfunction [1, 2]. The incidence is around one to four cases per million people per year, affecting all age groups with a bimodal peak of 10 to 19 years and 30 to 39 years [3]. The pathogenesis remains unknown; however, some mechanisms include focal inflammation and vascular damage of the spinal cord, frequently resulting in significant morbidity and mortality [3]. As proposed by the Transverse Myelitis Consortium Working Group from Johns Hopkins in 2002, the diagnosis of TM is made by the presence of clinical manifestations attributable to spinal cord damage, a clearly defined sensory level, and evidence of inflammation on magnetic resonance imaging (MRI) consistent with myelitis [1]. TM occurs in autoimmune connective tissue disorders including systemic lupus erythematosus (SLE), Sjogren's syndrome, and mixed connective tissue diseases as well as a variety of infectious diseases [3].

The prevalence of TM among SLE patients is estimated at 1–2%, which is 1,000 times greater than that reported for the general population [4–6]. Focal or extensive longitudinal lesions involving more than three contiguous vertebrae may occur, the latter being more frequent in SLE patients [7]. Gray and white matter involvement may occur [8]. Some pathophysiologic mechanisms include vasculopathy, autoantibody-mediated damage, proinflammatory cytokines, oxidative stress, and neurotransmission interference [9–12].

Environmental factors such as viral infections have been shown to play an important role in both SLE induction and exacerbations [13]. Viruses may alter innate and adaptative immune systems, consequently leading to dysregulation of the immune system that could translate into a variety of clinical manifestations and expressions of SLE [13]. Influenza viruses are RNA microorganisms that belong to the *Orthomyxoviridae* family [14]. There are four genera of influenza viruses of which type A is the most virulent among humans. It has been implicated in neurologic disorders, but only a few cases causing TM have been reported [15–17]. Furthermore, influenza vaccination has been associated as a possible cause of TM [18]. Herein, we report a patient with SLE who developed TM after influenza A virus infection.

## 2. Case Presentation

A 30-year-old Puerto Rican woman with SLE and hypothyroidism presented to the emergency department (ED) on May 4, 2019, with flu-like symptoms. Her SLE was diagnosed in 2010 when she presented with constitutional symptoms, photosensitivity, arthritis, serositis, nephritis, positive antinuclear antibodies (ANA), elevated anti-dsDNA antibodies, and C3 and C4 hypocomplementemia. Initially, she was treated with hydroxychloroquine and corticosteroids. Mycophenolate mofetil was added in 2013 for better control of lupus nephritis. She responded well to this treatment; proteinuria and hematuria almost resolved, and her renal function remained normal. On evaluation at the ED, she was taking prednisone 5 mg daily, hydroxychloroquine 300 mg daily, and mycophenolate mofetil 1000 mg twice daily.

On May 1, 2019, she developed general malaise, fever, headaches, myalgias, arthralgias, nausea, vomiting, anorexia, and nonbloody diarrhea associated with a skin rash over the arms and chest. Three days later, her symptoms worsened, and she was found with a positive type A influenza test at a laboratory facility for which she went to the ED. The next day, while in the ED, she experienced low back pain associated with sudden urinary retention followed by bilateral lower extremity weakness. She rapidly deteriorated over the next 24 hours and the next day, she was unable to walk by herself and developed distal upper extremity weakness, decreased perianal and bilateral lower extremity sensation, and bilateral blurred vision more prominent in the left side.

On initial examination, the temperature was 36°C, the blood pressure was 92/48 mm Hg, and the heart rate was 105 beats per minute. Physical examination was normal except for a hard palate ulcer and a macular papular skin rash over the chest and upper extremities. Twenty-four hours later, she developed marked bilateral lower extremity weakness, decreased handgrip strength, and anal wink. Sensation was decreased in the lower extremities and in the perianal region. Deep tendon reflexes were increased in the upper and lower limbs. Ophthalmologic examination demonstrated left eye retinal detachment associated with bilateral optic nerve and macular edema. Pulmonary, cardiac, abdominal, and articular examinations were normal.

**Figure 1:**
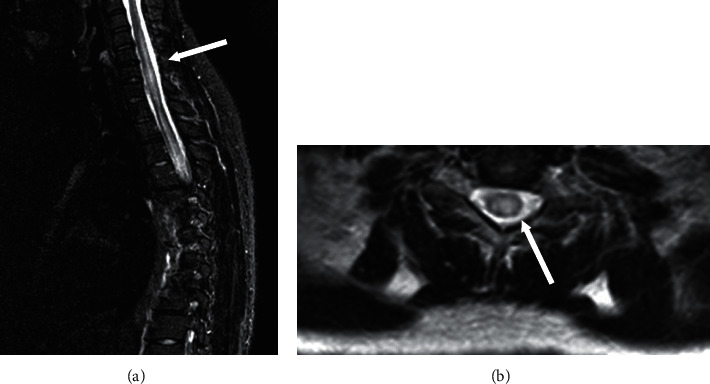
Initial MRI of the cervical and thoracic spine. (a) Sagittal short tau inversion recovery (STIR) image shows increased signal intensity of the spinal cord beginning at C4 with caudal extension into the thoracic spine. (b) T2-weighted axial image at the C4 level demonstrates increased central signal intensity of the spinal cord.

Initial laboratory tests revealed leukopenia (3.1 × 10^9^/L), lymphopenia (0.28 × 10^9^/L), and anemia (9.8 g/dl), but a normal platelet count (147 × 10^9^/L). Serum creatinine levels were normal at 0.9 mg/dl. She had hypoalbuminemia (2.7 g/dL), but normal total bilirubin (0.23 mg/dL), alkaline phosphatase (61 mg/dL), aspartate aminotransferase (30 mg/dL), and alanine aminotransferase (17 mg/dL). Lipase and amylase serum levels were normal. Urinalysis showed proteinuria (75 mg/dL) and granular casts (0–2/high power field (hpf)) without microscopic hematuria (0–3 RBC/hpf) or pyuria (0–4 WBC/hpf). Spot urine showed a protein-to-creatinine ratio of 1.33. The Westergren erythrocyte sedimentation rate (ESR) was markedly elevated at 113 mm/h, and the C-reactive protein (CRP) was increased (10.3 mg/L; normal range <5 mg/L). She had elevated anti-dsDNA (1 : 80), anti-RNP (11 IU/mL; normal range <5 IU/mL), and IgA anti-B2-glycoproteins I antibodies (38 SAU, normal range <20 SAU), and C3 (33.5 mg/dL; normal range: 90–180 mg/dL) and C4 (3.3 mg/dL; normal range 10–40 mg/dL) hypocomplementemia. The lupus anticoagulant test and anti-Ro, anti-La, anti-Smith, anti-cardiolipin (IgA, IgM, and IgG), anti-B2-glycoprotein I (IgM and IgG), antineutrophil cytoplasmic, and anti-aquaporin-4 (AQP4) antibodies were negative. The nasopharyngeal antigen test for influenza type A was positive. Influenza type B, human immunodeficiency virus (HIV), rapid plasma reagin (RPR), cytomegalovirus (CMV), hepatitis B and hepatitis C, and mycoplasma tests were negative. Blood, urine, and stool cultures were negative. Brain magnetic resonance imaging (MRI) was normal. MRI of the spinal cord showed a long hyperintense lesion from C4 to T12 with predominant gray matter involvement (Figure 1) and a smaller area of thickening of the conus medullaris with central cord abnormal signal intensity from T12 to L1.

Upon arrival to the ED, she was treated with oseltamivir 75 mg orally twice daily for 5 days. On May 7, 2019, she was started on intravenous (IV) methylprednisolone 1,000 mg daily for 3 days and received IV cyclophosphamide 850 mg. Laboratory tests on the day of initial cyclophosphamide therapy revealed persistent leukopenia (3.0 × 10^9^/L), lymphopenia (0.50 × 10^9^/L), and anemia (9.8 g/dl), but a normal neutrophil count (1.7 × 10^9^/L) and platelet count (149 × 10^9^/L). IV immunosuppressive treatment was followed by oral prednisone at 1 mg/kg. She was continued on hydroxychloroquine 300 mg daily, but mycophenolate mofetil therapy was held. Five days after the initiation of therapy, upper extremity strength, perianal sensation, and bilateral lower extremity sensation started to improve. Nonetheless, she continued with marked bilateral lower extremity weakness. The eye exam exhibited mild improvement but persisted with optic nerve and macular edema. On May 15, 2019, she was transferred to an in-patient rehabilitation center.

**Figure 2:**
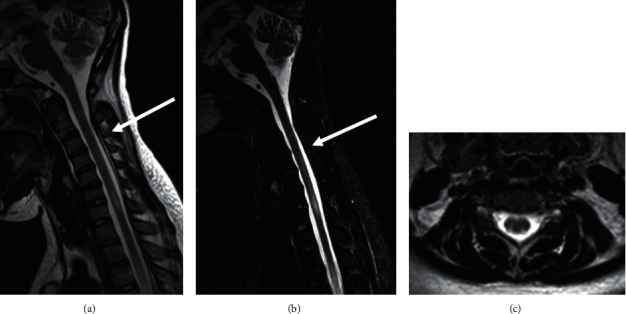
Follow-up MRI of the cervical and thoracic spine two months after treatment. (a) Sagittal STIR and (b) T2-weighted MRI images show interval improvement of the previously seen abnormal spinal cord signal intensity. (c) Axial T2-weighted image at C4 level demonstrates interval improvement of the previously seen abnormal signal intensity.

One month after discharge, she was continued on monthly IV cyclophosphamide, oral prednisone and hydroxychloroquine, and mycophenolate mofetil 1000 mg twice daily was restarted. The prednisone dose was initially decreased to 40 mg daily. She had no clinical evidence of systemic manifestations and her neurologic motor deficits and visual acuity gradually improved. She still needed a wheelchair for mobilization. Laboratory tests revealed no leukopenia, lymphopenia, or thrombocytopenia. Serum creatinine level was normal (0.46 mg/dL), but serum albumin levels remained low (2.7 g/dL). Liver enzyme tests were normal and ESR decreased to 74 mm/hr. Two months later, a follow-up spinal cord T2-weighted MRI showed interval improvement in the degree of abnormal signal intensity contrast enhancement lesions abovementioned (Figure 2).

Six months after discharge, she completed six cycles of IV cyclophosphamide (850 mg monthly). She continued maintenance therapy with hydroxychloroquine, mycophenolate mofetil, and prednisone dose which was gradually tapered to 10 mg daily. At that time, her motor dysfunction almost resolved, and she was able to walk with a walker. She had mild residual weakness in her right lower extremity. Visual acuity returned to baseline. She had mild anemia (10.5 g/dL), but no leukopenia, lymphopenia, or thrombocytopenia. Serum creatinine (0.53 mg/dL) and albumin (4.2 g/dL) levels were normal. Urinalysis was normal, and spot urine for protein and creatinine showed a ratio of 0.23. ESR decreased to 22 mm/h. Anti-dsDNA antibodies and C3 and C4 complement levels were normal. The prednisone dose was tapered to 5 mg orally daily, and she continued maintenance therapy with mycophenolate mofetil. After a follow-up period of 28 months, her SLE remained stable and did not develop any further SLE exacerbations. Regarding neurological symptoms, she was able to walk without assistance, although she remained with minimal gait disturbances due to right distal lower extremity weakness.

## 3. Discussion

We report a patient with SLE who developed extensive longitudinal TM following an influenza A virus infection who was successfully treated with high-dose corticosteroids and IV cyclophosphamide. Neurologic manifestations are reported in up to 60% of patients with SLE [5, 19]. The most common manifestations are cerebrovascular accidents, seizures, and peripheral neuropathy. TM induced either by SLE or influenza is very uncommon; furthermore, the occurrence of both conditions is extremely rare. In fact, no previous cases in the literature have described TM in an SLE patient having an influenza A virus infection. Certainly, the coexistence of these conditions has significant clinical and prognostic implications, as described in this case.

Neurological manifestations associated with influenza A virus infection have been previously described. The most common complications reported are encephalitis, seizures, Guillain–Barre syndrome, Reye's syndrome, and Parkinsonian manifestations, and less frequently, acute disseminated encephalomyelitis, Klein–Levin syndrome, and transverse myelitis [16, 20–24]. TM triggered by influenza A virus has been described most commonly in children, frequently resulting in severe neurological sequelae and lethal outcomes [16, 21]. Interestingly, some studies have described cases of TM induced by the vaccine for the 2009 subtype of H1N1 influenza A [25, 26]. Our patient lacked respiratory manifestations which is in contrast with previous case reports of influenza-mediated TM [22, 24]. Also, neurologic symptoms rapidly progressed in our patient within five days of viral symptoms onset. The average time seen in other case reports is approximately ten days from the onset of influenza A viral symptoms to the development of neurologic symptoms [22–24]. On the other hand, symptoms of postvaccination demyelinating diseases occur at a mean of 14.2 days after immunization [22].

In patients with SLE, cases of TM are usually seen during the early stages of lupus when patients exhibit higher levels of disease activity [27]. In our case, TM occurred nine years after the diagnosis of SLE. Prior to TM, her SLE was well controlled with hydroxychloroquine, low-dose prednisone, and mycophenolate mofetil. Thus, influenza type A was the most likely culprit of TM. It is not unexpected that there is a link between SLE, TM, and influenza A. There is a robust evidence that influenza A virus may induce dysregulation of the immune system through several mechanisms including increased expression of interferon-*α* (IFN- *α*), tumor necrosis factor-*α*, IL-1, Il-6, and Il-8, and monocyte attracting chemokines [28]. Among these cytokines, IL-6 and IL-8 are known to upregulate MMP-9, which has been found significantly elevated in patients with neuropsychiatric SLE when compared to those without central nervous system involvement [29]. A correlation with MMP-9 and neuronal and glial degradation products was found in this study. In addition, it is well known that in SLE endogenous Toll-like receptor (TLR) ligands such as DNA- or RNA-containing particles generated from apoptosis can trigger the innate immune system and stimulate IFN-*α* production [30–32]. Recent evidence shows that viral infections could contribute to this dysregulated pathway by increasing the expression of TLR9 in B cells and monocytes and further activation of TLRs and IFN systems [13].

As in our patient, extensive longitudinal TM is frequently reported in SLE. A retrospective study of 22 patients with SLE and TM described gray and white matter spinal lesions [14]. Patients with central gray matter lesions presented with weakness associated to flaccid paralysis and hyporeflexia, in contrast to those with outer white matter lesions who manifested with spasticity and hyperreflexia. TM in patients with gray matter lesions occurred more commonly in the context of highly active SLE and was associated with extensive longitudinal TM, rapid progression of neurologic symptoms, resistance to treatment, and more disability than those with white matter lesions. As seen in our patient, gray matter clinical manifestations were associated with urinary retention prior to irreversible paraplegia and the onset of neurologic symptoms that were preceded by fever, nausea, and vomiting. In contrast, our patient had a good clinical response, despite presenting with a subtype associated with worse clinical outcome.

A reasonable concern for our patient is whether she would benefit or not from influenza vaccination. Issues about vaccines triggering autoimmune demyelinating diseases have been extensively portrayed in the literature [33–35]. As noted before, some case reports have described TM induced by the vaccine for the 2009 subtype of H1N1 influenza A [25, 26]. We acknowledge the benefits of vaccination in preventing viral disease and complications, including respiratory and neurological involvement in patients with autoimmune diseases such as SLE, particularly those exposed to immunosuppressive therapy. Unfortunately, limited research has evaluated the impact of influenza vaccination specifically in patients with previous neurological complications [36, 37]. No studies have been published evaluating vaccination outcomes in patients with TM. Nonetheless, a retrospective study in patients with neuromyelitis optica spectrum disorders disclosed nine cases of relapses associated with influenza vaccination, but none of these patients were receiving immunosuppressive therapy [38]. However, the same study showed lower relapse rates in patients who received vaccinations when compared to those who did not. It is well known that SLE patients are at an increased risk of viral infections; thus, vaccination may help prevent infection and related complications. Special considerations should be made in patients with active SLE as vaccine mechanisms may induce autoimmunity and therefore might trigger an SLE flare. In such cases, withholding vaccination might be the safest; nevertheless, further studies to assess these implications are needed for better understanding.

To the best of our knowledge, we report the first case of TM after influenza A infection in a patient with SLE. Clinicians should be aware that viral infections, including influenza, may induce TM. The favorable clinical outcome observed in our patient highlights the importance of early recognition and prompt initiation of immunosuppressive therapy.
